# Current Challenges and Future Prospects in Human Reproduction and Infertility

**DOI:** 10.3390/medicina60101627

**Published:** 2024-10-05

**Authors:** Laurie Henry, Michelle Nisolle

**Affiliations:** 1 Center for Reproductive Medicine of the University of Liège, Boulevard du 12ème de Ligne 1, 4000 Liege, Belgium; 2 Obstetrics and Gynecology Department, University of Liège, Boulevard du 12ème de Ligne 1, 4000 Liege, Belgium; michelle.nisolle@citadelle.be

Human reproduction is a captivating yet intricate field, constantly presenting new challenges and discoveries. Despite the remarkable progress made in assisted reproductive technologies (ARTs)—from intrauterine insemination to in vitro fertilization (IVF), with or without pre-implantation genetic testing—many obstacles remain. Failures in ART can be emotionally taxing, financially burdensome, and physically demanding for the individuals involved. Two key processes—genetic recombination during fertilization and embryo implantation in the endometrium—remain only partially understood. Improving ART outcomes is a top priority for reproductive specialists who are committed to optimizing success while minimizing the associated burdens on patients and ensuring positive long-term outcomes for their future children.

In addition to enhancing ART outcomes, it is crucial to address infertility prevention. This issue is gaining importance as the average age of first conception continues to rise, exposing individuals to a variety of environmental and lifestyle factors that can negatively influence fertility. At the same time, we face the challenge of safeguarding fertility against the potentially harmful effects of medical treatments, such as chemotherapy, and surgical procedures, like ovarian surgery, which can pose risks to the ovarian reserve, particularly in cases involving ovarian endometriosis [1,2].

This Special Issue of *Medicina* addresses these pressing challenges and offers insights into the future of reproductive medicine. It includes 14 studies that provide in-depth perspectives on current challenges in the field. These contributions span a range of topics, including ovarian reserve preservation, fertility prevention strategies, recurrent pregnancy loss (RPL), repeated implantation failure (RIF), advancements in ovarian stimulation and IVF techniques, and long-term ART effects. Each study contributes to a broader understanding of how we can enhance reproductive care and outcomes for patients facing infertility.

Several studies in this Special Issue focus on preserving the ovarian reserve in the face of medical interventions. For example, Soner Gök and colleagues examine the impact of selective serotonin reuptake inhibitors (SSRIs), commonly used for depression, on the ovarian reserve. Their research shows significant reductions in anti-Müllerian hormone (AMH) levels and antral follicle count (AFC) following six months of SSRI treatment in women with major depressive disorder [3]. Their study highlights the potential risks of SSRIs on reproductive health and underscores the need for fertility awareness before initiating such treatments.

In the study by Jules Bindels, ovarian tissue cryopreservation (OTC) is explored as a promising fertility preservation option, particularly for prepubertal patients or women unable to undergo oocyte cryopreservation. We propose using rapamycin, an mTOR inhibitor, to inhibit follicle activation, potentially extending the lifespan of ovarian grafts after autotransplantation [4]. This innovative approach could significantly improve the success rates of future fertility preservation. Further research is needed to fully explore and validate this possibility.

In the field of in vitro fertilization (IVF), continued advancements in ovarian stimulation protocols and lab techniques are key to improving outcomes, especially for patients facing challenging conditions such as endometriosis, advanced maternal age, or unexplained infertility.

This Special Issue also covers the complex intersection of endometriosis and IVF, a topic addressed in a comprehensive review by Sania Latif. Her work delves into the unique challenges that endometriosis presents to ART, specifically its adverse effects on oocyte quality and endometrial receptivity. Despite advancements in IVF techniques, endometriosis continues to impair success rates. Its review emphasizes the importance of individualized treatment strategies, including tailored stimulation protocols to optimize outcomes for these patients [5].

Maternal age and its impact on ART success are explored in a study by Jelena Havrljenko et al., who analyze the optimal number of metaphase two (MII) oocytes needed to achieve a live birth in women of advanced maternal age. Their findings suggest that clinicians should aim to retrieve at least nine MII oocytes during IVF to maximize live birth rates (LBRs) in women over 40, though success rates decline significantly after the age of 42 [6].

Inflammation and oxidative stress are major factors affecting IVF outcomes, and a pilot study by Renato Colognato and his team investigates the potential benefits of a Curcuma-based supplement (NOFLAMOX) in reducing these factors. The results are promising, with significantly improved pregnancy rates in patients treated with the supplement, demonstrating the potential role of immune modulation in enhancing ART outcomes [7].

Another promising approach in ovarian stimulation is presented by Bulut Varlı et al., who evaluate the use of letrozole combined with a GnRH antagonist protocol in patients with poor ovarian response. While the addition of letrozole did not lead to significantly higher live birth rates, it showed promise in reducing gonadotropin use and shortening stimulation duration, providing a more cost-effective option for patients with a diminished ovarian reserve [8].

Lastly, our own research team has made strides in optimizing the use of monopronuclear (1PN) zygotes in IVF. Historically considered abnormal, 1PN zygotes are often discarded. However, our retrospective analysis revealed that these zygotes can develop into viable embryos, leading to successful pregnancies and healthy live births [9]. This research finding opens the door to new possibilities in embryo selection and may help improve success rates in cases where conventional methods fall short.

Moving to the topic of RPL and RIF, Borsi et al. investigate the role of genetic mutations linked to thrombophilia, such as Factor V Leiden (FVL G1691A) and Methylenetetrahydrofolate Reductase (MTHFR C677T), which are shown to increase the risk of miscarriage. Their findings underscore the importance of genetic testing in recurrent pregnancy loss and the potential benefits of targeted treatments [10].

In a separate study on immunological factors, Seles et al. evaluate the impact of Killer-cell Immunoglobulin-like Receptors (KIRs) on reproductive outcomes in RIF patients. Their results suggest that certain genotypes are associated with improved pregnancy outcomes when patients receive immunomodulatory treatments, highlighting the potential for personalized immune therapies in reproductive medicine [11].

Among the rare conditions contributing to infertility is endometrial osseous metaplasia, as discussed by Tica et al., who describe a case of primary infertility resulting from bone tissue in the endometrium. After successful removal, the patient achieved a spontaneous pregnancy, underscoring the need to consider uncommon conditions in fertility diagnostics [12].

Further innovation comes from the work of Kushniruk et al., who explore the use of granulocyte colony-stimulating factor (G-CSF) in patients with RIF undergoing oocyte donation. While their results are not statistically significant, their study points to G-CSF’s potential in enhancing endometrial receptivity and improving implantation rates [13].

Finally, Kitaya et al. addressed the diagnosis of chronic endometritis (CE), a condition linked to unexplained infertility, endometriosis, and RIF. Typically diagnosed through invasive biopsy, their team developed a deep learning model using hysteroscopic imaging to detect CE. The ARCHIPELAGO study aims to offer a less invasive, more comprehensive diagnostic method, potentially shedding new light on a cause of recurrent pregnancy loss and implantation failure [14].

In terms of long-term outcomes, Collée et al. evaluate the maternal and child health risks associated with ART. Their study reveals higher rates of pre-eclampsia and intensive care unit admissions among women who conceived through ART, particularly after frozen embryo transfers. These findings highlight the importance of continuous monitoring for both maternal and fetal health in ART pregnancies [15].

Building on long-term outcomes, Moustakli and Tsonis explore the effects of gender-affirming hormone therapy on reproductive health in transgender individuals. While crucial for mental health, hormone therapy may increase risks like reduced bone density and cardiovascular issues. They emphasize the need for fertility preservation options and further research into the long-term reproductive effects, advocating affirming, comprehensive care [16].

In conclusion, the 14 studies featured in this Special Issue of *Medicina* collectively advance our understanding of infertility, ART, and reproductive health. They underscore the need for personalized, patient-centered approaches that take into account individual risk factors, emerging therapies, and the long-term effects of ART. Whether through ovarian reserve preservation, immune modulation, or optimizing IVF protocols, these studies pave the way for improved outcomes and more compassionate care in reproductive medicine.
